# Bone mineral content in growth hormone deficient children treated with growth hormone after withdrawal of 1 year of supplementation with calcium, vitamin D and zinc

**DOI:** 10.1186/1687-9856-2015-S1-P117

**Published:** 2015-04-28

**Authors:** Vaman Khadilkar, Veena Ekbote, Shashi Chiplonkar, Anuradha Khadilkar

**Affiliations:** 1 Hirabai Cowasji Jehangir Medical Research Institute, Jehangir Hospital, Pune, Maharashtra, India

## 

Supplementation with calcium (ca), vitamin d (vit D), zinc has been shown to have a positive effect on bone mineral content (BMC) gain in growth hormone deficient (GHD) children on GH therapy[1]. It is unknown if this gain is sustained after supplement withdrawal. We aimed to investigate the influence of prior supplementation with ca, vitD and zinc on BMC accretion after supplement withdrawal.

31 prepubertal GHD children were randomly allocated to receive A) calcium (500mg/d), vitamin d (30,000 IU/3 months) and B) calcium (500mg/d), vitamin D (30,000 IU/3 months) & zinc (8 mg) for 1 year with GH. Ht measurement, bone mineralization by dual energy x-ray absorptiometry, tanner staging were performed at 4 timepoints, baseline, post 1 year of supplementation and 1 & 2 years after withdrawal of supplementation. Height for age z-scores (HAZ) were calculated from ethnic growth references.

At baseline, children (18 boys, 9.6±2.8 years) from group A & B were similar in their HAZ (-4 ±1.5, -4 ±1.3) and BMC (370±215 g, 440±167g). 1 year post supplementation, 40% & 36% children and by the end of 2^nd^ year of supplementation withdrawal, 47% & 80% from group A & B respectively had entered puberty. Since Ht has strong correlation with BMC, % change in ht adjusted BMC was analyzed. The gain in BMC was greater (p < 0.05) in group B (51 %) children than in group A (49 %) children^2^. However, after the withdrawal of the supplementation, the % gain in ht as well as ht adjusted BMC was similar in both groups. The % gain in ht adjusted BMC was lower (p <0.05) in the 1^st^ year of supplement withdrawal (22 %). In 2^nd^ year, the ht adjusted BMC showed a significantly greater (53 %, p < 0.05) gain than the supplementation year and first year after supplementation withdrawal.

Effect of short term supplementation with ca, vit D & zn in GH treated GHD children may not continue after the withdrawal of supplementation. However, the greater gain in the 2^nd^ year after supplementation withdrawal was possibly due to the effect of puberty.

## References

[B1] Ekbote V Khadilkar A Chiplonkar S Mughal Z Khadilkar V Enhanced effect of zinc and calcium supplementation on bone status in growth hormone-deficient children treated with growth hormone: a pilot randomized controlled trial. Endocrine 2013 43 3 686 95 10.1007/s12020-012-9847-0 23224626

